# A case of torsades de pointes induced by the third-generation EGFR-TKI, osimertinib combined with moxifloxacin

**DOI:** 10.1186/s12890-020-01217-4

**Published:** 2020-06-24

**Authors:** Shuang Bian, Xiaomiao Tang, Wei Lei

**Affiliations:** Department of Pulmonary and Critical Care Medicine, The First Affiliated Hospital of Soochow University, Suzhou, 215006 Jiangsu China

**Keywords:** TdP, QT interval, Adverse events, EGFR-TKIs, Osimertinib, Moxifloxacin

## Abstract

**Background:**

Torsade de pointes (TdP) is a malignant arrhythmia that can be induced by QT internal prolongation due to a variety of factors. Here we report an elderly patient with advanced non-small cell lung cancer (NSCLC) had sudden TdP during hospitalization, which was caused by multiple factors such as osimertinib, moxifloxacin and patient self-factors.

**Case presentation:**

An 85-year-old man with advanced NSCLC with brain andbone metastasis was initially treated with gefitinib targeted therapy. After 4 months treatment, the patient developed drug resistance and a second genetic testing revealed that the T790M mutation was positive. And the patient was then changed to targeted therapy with osimertinib, followed by adverse reactions of varying severity such as diarrhea, electrolyte imbalance, decreased cardiac function, leukopenia, and prolonged QTc interval. Six months after the administration of osimertinib, the patient was admitted to the hospital, chest CT showed the lesion progressed again, and during which hospital-acquired infection occurred. After concomitant use of moxifloxacin, the patient had sudden TdP, and finally died of this cardiac event.

**Conclusions:**

It is suggested that clinicians need to identify patients with high risk factors of TdP, and consider comprehensively in concomitant medication to avoid such events to the greatest extent.

## Background

Torsade de pointes (TdP) is a malignant arrhythmia that can be induced by QT internal prolongation due to a variety of factors, such as age, gender, heart rate, electrolytes, drugs, cardiac diseases, central nervous system diseases, metabolic diseases, infectious diseases, tumors, and fever [1, 2]. Here we present an elderly patient with advanced non-small cell lung cancer (NSCLC) had sudden TdP during hospitalization, which was caused by multiple factors such as osimertinib, moxifloxacin and patient self-factors.

## Case presentation

An 85-year-old man who had a history of advanced lung adenocarcinoma for 1 year was admitted to our hospital with complaints of exacerbating cough and chest distress for 1 month on October 24, 2018. In September 2017, the patient visited our hospital due to cough, chest distress and fatigue. Chest CT: space-occupying lesions in the left upper lung, which was considered to be the lung cancer with obstructive pneumonia in the left upper lung; accompanied by left pleural effusion. Brain MRI: a left occipital lobe nodule, which was considered to be a metastatic tumor. PETCT: Partial rib and thoracic vertebra had higher levels of glucose metabolism, which might be the lung cancer bone metastasis. Then cytological examination of exfoliated cells in hydrothorax confirmed lung adenocarcinoma. The stage after assessment was T4N1M1c, stage IVB. Genetic testing showed that exon 21 L861Q of EGFR gene was positive, mutation frequency: 3.23%. The patient was given gefitinib (Iressa) 250 mg qd for targeted therapy. In February 2018, re-examination of chest CT showed progression of tumor lesions. In April 2018, a second genetic testing: exon 20 T790M of EGFR gene was positive. The treatment was adjusted to osimertinib (Teresa) 80 mg qd. Meanwhile, echocardiography was performed and showed the left ventricular ejection fraction (LVEF): 73%. Half a month after administration of osimertinib, the patient experienced alternating diarrhea and constipation, mainly diarrhea, the initial diarrhea was assessed as NCI-CTCAE V5.0 grade 1–2, and electrolyte imbalance was also observed: mild hypokalemia (potassium 3.0–3.5 mmol/L). The patient visited the outpatient for antidiarrheal and maintenance electrolyte therapy several times. During this period, the follow-up chest CT indicated that osimertinib treatment was effective, which was evaluated as partial response. When the patient was admitted to our hospital on October 24, 2018, the diarrhea was severe, which was assessed as NCI-CTCAE V5.0 grade 3.

**Fig. 1:**
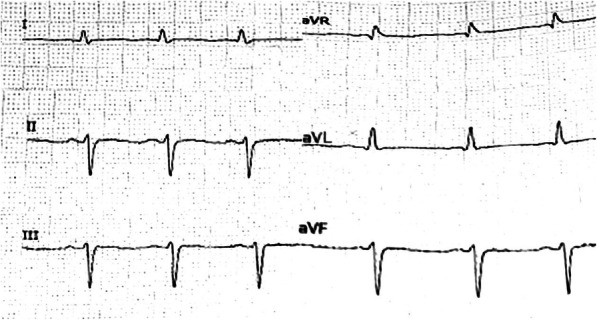
ECG on admission (Partial chest leads of bedside ECG were not completely recorded)

**Fig. 2:**
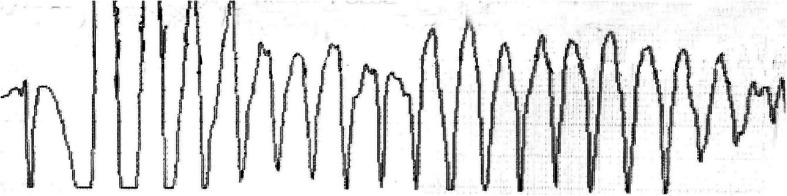
ECG monitoring showed TdP

The vital signs were T 36.0 °C, BP 99/64 mmHg, heart rate 86 beats per minute, and respiratory rate 20 breaths per minute during the initial examination. Chest CT on admission showed left upper lung tumor enlarged compared with that a month ago, with pleural effusion; the outcome of clinical efficacy evaluation was progressive disease; electrolytes: potassium 2.94 mmol/L; blood routine: white blood cells 1.16 × 10^9^/L, neutrophils 0.56 × 10^9^/L, hemoglobin 78 g/L, platelets 82 × 10^9^/L; NT-proBNP: 5221 pg/mL; echocardiography: LVEF: 41%; Electrocardiogram (ECG): sinus rhythm, left anterior fascicular block, QTc interval 484 ms (Fig. 1). Then thoracentesis was performed for pleural effusion, biapenem preventive anti-infection, recombinant human granulocyte colony-stimulating factor (rhG-CSF) to stimulate hematopoietic system, antidiarrheal, potassium supplement and other treatments. Electrolytes after potassium supplementation: potassium 3.75 mmol/L. In addition, considering severe diarrhea, neutropenia and other adverse reactions, the administration of osimertinib was temporarily stopped. On the 3rd day after admission, the patient had a fever with a peak temperature of 39.1 °C, and was given Moxifloxacin Hydrochloride and Sodium Chloride Injection (Avelox, specification: 250 ml: 0.4 g) ivgtt. The infusion time was about 100 min. Twenty minutes after the end of infusion, ECG monitoring showed TdP (Fig. 2), and the patient had transient syncope. ECG: QTc interval 647 ms. Magnesium supplementation, potassium supplementation, and the antiarrhythmic drug lidocaine were given for emergency treatment. The patient did not have recurrent TdP afterwards. ECG after 7 h: QTc interval 631 ms; ECG after 10 h: QTc interval 578 ms; ECG after 43 h: QTc interval 531 ms; ECG after 91 h: QTc interval 496 ms. On October 30, 2018, the patient experienced decreased blood pressure and pulse oxygen, and was unconscious. In order to relieve the patient’s pain, the family did not consider the tracheal intubation, chest compression or other invasive rescue measures, and the patient was discharged.

## Discussion and conclusions

The QT interval varies between individuals, and is influenced by various factors such as age, gender, heart rate, electrolytes, drugs, cardiac diseases, central nervous system diseases, metabolic diseases, infectious diseases, tumors, and fever [1, 2]. Since the value of QT interval is affected by the heart rate, Bazetts formula calculation is commonly used to convert it into a heart rate-independent correction value, that is, QTc interval (QTc = QT/RR^0.5^. The RR interval is a normalized heart rate value obtained by dividing 60 by heart rate). In a scientific statement from the American Heart Association and the American College of Cardiology Foundation published in 2010, the normal QTc interval is recommended to be 470 ms for males and 480 ms for females. QTc interval > 500 ms is considered highly abnormal in both males and females. The risk of developing TdP increases with QTc being prolonged. TdP is a malignant arrhythmia with clinical manifestations characterized by syncope, tetany, or sudden death. It usually terminates spontaneously, and in a few cases it further deteriorates and evolves into ventricular fibrillation, causing sudden cardiac death [1].

Epidermal growth factor receptor-tyrosine kinase inhibitor (EGFR-TKI) has been widely used in NSCLC patients with epidermal growth factor receptor (EGFR) mutation. Osimertinib, the third-generation EGFR-TKI that can specifically bind mutated T790M, has been approved for the treatment of NSCLC patients with T790M mutation [3–6]. Osimertinib has a good effect on brain metastasis [4, 7]. This patient had advanced NSCLC with brain and bone metastases. Besides, the T790M mutation was detected after progression of gefitinib treatment. Therefore the subsequent treatment regimen was adjusted to osimertinib. EGFR-TKIs can cause a variety of adverse reactions such as rash, digestive system toxicity, interstitial lung disease, oral ulcers, liver function damage, and ocular toxicity [3–6, 8].

In this case, the patient developed severe diarrhea during the administration of osimertinib. The exact mechanism by which EGFR-TKIs cause diarrhea is not fully understood, and EGFR is expressed on gastrointestinal epithelial cells. One of the hypotheses is that intestinal chloride secretion is the likely mechanism [9]. In the FLAURA study, a phase III clinical study of osimertinib, the incidence of diarrhea was comparable between the osimertinib group (osimertinib 80 mg qd) and the standard EGFR-TKI group (gefitinib 250 mg qd or erlotinib 150 mg qd) (58% vs 57%), which were mostly grade 1 (43% vs 42%) and grade 2 (both 13%). The incidence of severe grade 3 diarrhea adverse reactions in both group was low (both 2%) [4]. This patient had no obvious diarrhea symptom during the administration of gefitinib, but had severe diarrhea after switching to osimertinib for treatment, and the NCI-CTCAE V5.0 grade 3 was assessed, which might be related to different individual responses to varying EGFR-TKIs. Severe diarrhea, poor appetite and reduced food intake caused by tumor disease and drug of this patient could cause repeated electrolyte imbalance, leading to hypokalemia and hypomagnesemia. In addition, EGFR-TKIs themselves have the potential to contribute to the development of hypomagnesemia [8, 10]. Hypokalemia has been identified as a risk factor for TdP, which inhibits rapidly activating delayed rectifier potassium current (I_Kr_) in cardiomyocytes, prolongs QTc interval, results in heterogeneity and dispersion of repolarization, and predisposes to TdP. A similar picture is found for hypomagnesemia [1].

EGFR-TKIs themselves have the adverse effect of QTc interval prolongation. The incidence of adverse events of QTc prolongation caused by osimertinib was not high, and most of them were mild to moderate (grade 1–2) adverse events [4–6]. No events of arrhythmia were reported in two global single-arm clinical trials (AURA Study Phase II Extension Component and AURA2 study) [5, 6]. Although adverse events of QTc prolongation caused by osimertinib are rare, regular electrocardiograms are recommended in patients taking osimertinib. Dose reduction or treatment interruption should be performed for serious adverse events of QTc prolongation or even serious arrhythmia [6].

In the echocardiography examination of this patient, a decrease in LVEF was observed in only half a year, and the absolute value of the decrease reached 32%. EGFR-TKIs are associated with a risk of adverse effects on left ventricular dysfunction with low occurrence, which develops from asymptomatic electrocardio changes to left ventricular functional decline and to severe heart failure and fluid retention [11]. Decrease in ejection fraction can be detected by echocardiography [12]. Currently available studies are not able to determine a causal relationship between osimertinib and changes in cardiac contractility. Pathophysiological changes such as cardiac insufficiency can cause abnormal cardiac repolarization, combined with other risk factors, which will aggravate QTc interval prolongation and induce TdP. Therefore, assessment of cardiac function is recommended before and during EGFR-TKI therapy to facilitate early detection of drug-related adverse events.

EGFR-TKIs can cause hypocytosis with a high frequency. The blood routine examination showed pancytopenia in this patient. This patient had advanced bone metastasis of lung cancer, and factors such as malnutrition and bone marrow infiltration of tumor cells might cause pancytopenia. However, it was not excluded that adverse reactions of EGFR-TKIs were involved in the occurrence of pancytopenia. In addition, long-term diarrhea of the patient led to the digestive disorder and undernutrition, which also resulted in the immune dysfunction. Infection and fever caused by immunocompromise in this patient added the risk factors for QTc prolongation [1].

Moxifloxacin is a fourth-generation broad-spectrum fluoroquinolone antibiotic. The adverse reactions of moxifloxacin are similar to those of other quinolones. However it causes a higher risk of adverse reactions of QTc prolongation than other quinolones (ciprofloxacin, levofloxacin, ofloxacin, etc.) and is more likely to cause severe arrhythmias [13, 14]. Rapid intravenous infusion causes an increase in plasma concentrations, bringing the higher risk of TdP [1, 2]. Moxifloxacin-induced QTc prolongation occurs in a concentration-dependent manner [15]. Therefore, the recommended dose and drip rate (infusion rate of 0.4 g in 90 min) should not be exceeded. Some scholars proposed that antibiotics such as quinolones could be appropriately used for treatment when dealing with the adverse reactions of EGFR-TKIs, if severe diarrhea lasts for more than 24 h accompanied by fever or neutropenia [8]. However, in this case, the occurrence of this cardiac event might be induced by quinolones.

For this patient, although we conducted the treatment such as rhG-CSF to stimulate the production of white blood cells, biapenem preventive anti-infection, the fever occurred on the 3rd day after admission, which was considered to be hospital-acquired infection. The patient was an elderly cancer patient with leukopenia, belonging to the population susceptible to hospital-acquired infection [16]. After the patient developed fever, moxifloxacin was added without considering the patient’s multiple risk factors for arrhythmia, and although electrolytes were corrected after admission, the adverse event eventually occurred. Therefore, the use of quinolones, especially those given intravenously easy to cause arrhythmia, should be particularly cautious for patients with risk factors such as advanced age, underlying cardiac disease, and electrolyte imbalance in clinical practice.

To our knowledge, this is the first report on TdP induced by osimertinib combined with moxifloxacin. As to this patient with advanced NSCLC, adverse reactions such as diarrhea, electrolyte imbalance, decreased cardiac function, leukopenia, and prolonged QTc interval occurred during the treatment with osimertinib. Besides he developed hospital-acquired infection after hospitalization, and underwent TdP after the moxifloxacin was added. It suggests that clinicians need to identify patients with high risk factors of TdP, and consider comprehensively in concomitant medication to avoid such events to the greatest extent.

## Data Availability

The data set supporting the results of this article are included within the article.

## Abbreviations

TdP: Torsade de pointes
NSCLC: Non-small cell lung cancer
LVEF: Left ventricular ejection fraction
ECG: Electrocardiogram
NCI-CTCAE V5.0: National Cancer Institute, Common Terminology Criteria for Adverse Events Version 5.0
EGFR: Epidermal growth factor receptor
EGFR-TKI: Epidermal growth factor receptor-tyrosine kinase inhibitor
rhG-CSF: Recombinant human granulocyte colony-stimulating factor
